# StarD13 is a tumor suppressor in breast cancer that regulates cell motility and invasion

**DOI:** 10.3892/ijo.2014.2330

**Published:** 2014-03-07

**Authors:** SAMER HANNA, BASSEM KHALIL, ANITA NASRALLAH, BECHARA A. SAYKALI, RANIA SOBH, SELIM NASSER, MIRVAT EL-SIBAI

**Affiliations:** 1 Department of Natural Sciences, The Lebanese American University, Beirut 1102 2801, Lebanon; 2 School of Medicine, The Lebanese American University, Beirut 1102 2801, Lebanon

**Keywords:** StarD13, RhoA, Rac, breast cancer, cell motility

## Abstract

Breast cancer is one of the most commonly diagnosed cancers in women around the world. In general, the more aggressive the tumor, the more rapidly it grows and the more likely it metastasizes. Members of the Rho subfamily of small GTP-binding proteins (GTPases) play a central role in breast cancer cell motility and metastasis. The switch between active GTP-bound and inactive GDP-bound state is regulated by guanine nucleotide exchange factors (GEFs), GTPase-activating proteins (GAPs) and guanine-nucleotide dissociation inhibitors (GDIs). We studied the role of StarD13, a recently identified Rho-GAP that specifically inhibits the function of RhoA and Cdc42. We aimed to investigate its role in breast cancer proliferation and metastasis. The levels of expression of this Rho-GAP in tumor tissues of different grades were assayed using immunohistochemistry. We observed that, while the level of StarD13 expression decreases in cancer tissues compared to normal tissues, it increases as the grade of the tumor increased. This was consistent with the fact that although StarD13 was indeed a tumor suppressor in our breast cancer cells, as seen by its effect on cell proliferation, it was needed for cancer cell motility. In fact, StarD13 knockdown resulted in an inhibition of cell motility and cells were not able to detach their tail and move forward. Our study describes, for the first time, a tumor suppressor that plays a positive role in cancer motility.

## Introduction

Breast cancer is one of the most commonly diagnosed cancers in women around the world. Ductal and lobular carcinomas are the two most frequent types of breast cancer. They can be either non-invasive, referred to as *in situ* carcinoma, or invasive infiltrating carcinoma (1). According to the US National Cancer Institute, breast cancer can be classified into five progressive stages. Stage 0 is referred to as carcinoma *in situ*, which can be either ductal carcinoma *in situ* (DCIS) or lobular carcinoma *in situ* (LCIS). DCIS may become invasive in later stages of the tumor and spread to other tissues (2,3). Invasive breast carcinoma can be classified into progressive stages I–IV depending on its size and its presence or absence at secondary sites, mainly the lymph nodes.

Cell motility is a complex multistep process that integrates multiple intracellular signaling and regulatory pathways. Therefore, slight modifications in any step may dramatically affect normal cellular functions and result in cellular transformation and carcinogenesis. It is known that cell motility is essential for metastasis and without it tumors would be easily eradicated and/or surgically removed (1). The acquisition of a motile phenotype is a critical step towards carcinogenesis and is required for a cell to gain metastatic competence. Thus, further descriptions of the molecular mechanisms regulating cancer cell motility would facilitate the development of specific and effective therapeutic treatments against metastasis and tumor cell invasion (1,4).

Members of the Rho-family GTPases are small GTP-binding proteins (GTPases) that range between 20–40 kDa in size. Almost all aspects of tumor cell proliferation, motility and invasion including cellular polarity, cytoskeletal re-organization, and signal transduction pathways are controlled through the interplay between the Rho-GTPases (5,6). Frequent studies have shown that the Rho family GTPases regulate cell motility in breast cancer through their ability to mediate the remodeling of the actin cytoskeleton as well as translating cellular signals from the plasma membrane receptors to regulate focal adhesion, cell polarity, vesicular trafficking and gene expression (6). Approximately 30% of human tumors possess a specific mutation in Ras oncogene leading to its protein level overexpression or constitutive activation. In contrast to Ras, no mutation in any of the Rho GTPases has been identified in breast cancer. Rather, these GTPases are often either overexpressed or hyperactive in breast cancer tissue. The variations in the levels of these Rho proteins might directly correlate with the advancement of breast cancer (7,8). The three most characterized members of the Rho GTPases are Rho, Rac and Cdc42 which were found to be distinct in function from the other Rho proteins (9). Rho GTPases are negatively regulated by Rho GTPases activating proteins (GAPs). These proteins inhibit Rho GTPases by activating their intrinsic GTPase activity. This leads to the hydrolysis of the bound GTP into GDP converting Rho GTPases back to their inactive conformation (10). In addition to activating GTP hydrolysis, GAPs may function as effectors of Rho GTPases to mediate other downstream effector functions (6,11)

*DLC2* gene was first identified by Ching *et al* (12). It is located on position *13q12.3* and was found to be underexpressed in hepatocellular carcinoma (12). DLC2 is commonly known as steriodogenic acute regulatory protein-related lipid transfer domain-containing protein 13 (StarD13). StarD13 shares 64% homology with DLC1, another member of the DLC family (13). StarD13 has an N-terminal SAM motif and a C-terminal START domain. It also harbors a RhoGAP domain, which is important to its function (12–14). Overexpression of StarD13 was found to associate with significant decrease in cell growth and proliferation in hepatocellular carcinoma (12). Moreover, DLC1, a closely related protein is found to be underexpressed in many types of cancer including lung, prostate, kidney, colon, breast, uterus and stomach (15). Also, previous data in astrocytoma suggest a potential role of StarD13 as a tumor suppressor (16).

In this study we aimed at characterizing StarD13 in breast cancer in terms of its level of expression and its role in cellular proliferation, motility and invasion. The level of expression of StarD13 was determined in patient tissues representing different grades of breast cancer compared to normal tissues. The effect on cellular proliferation, viability and cell cycle progression upon manipulating the level of StarD13 expression was then studied in addition to investigating its RhoGAP activity in cancer cell motility as well as its effect on cellular invasion *in vitro*.

## Materials and methods

### Cell culture

Human breast cancer cell lines (MCF-7 and MDA-MB-231) obtained from ATCC, were cultured in DMEM medium supplemented with 10% FBS and 100 U penicillin/streptomycin at 37˚C and 5% CO_2_ in a humidified chamber.

### Antibodies and reagents

Goat polyclonal anti-StarD13 antibody was obtained from Santa Cruz Biotechnology. Mouse monoclonal anti-RhoA, mouse monoclonal anti-Rac1, and mouse monoclonal anti-paxillin antibodies were purchased from Upstate Biotechnology (Lake Placid, NY, USA). Anti-goat and anti-mouse HRP-conjugated secondary antibodies were obtained from Promega. Fluorescent secondary antibodies (AlexaFluor 488) were obtained from Invitrogen. To visualize the actin cytoskeleton, cells were stained with Rhodamine phalloidin (Invitrogen). DAPI was also used to stain nuclei.

### Cell transfection with siRNA

Goat FlexiTube siRNA for StarD13, RhoA, and Rac1 were obtained from Qiagen. The siRNAs used had the following target sequences: StarD13: 5′-CCCGCAATACGCTCAGTTATA-3′, RhoA: 5′-TTCGGAA TGATGAGCACACAA-3′, and Rac1: 5′-ATGCATTTCCTG GAGAATATA-3′. The cells were transfected with the siRNA at a final concentration of 10 nM using HiPerfect (Qiagen) as described by the manufacturer. Control cells were transfected with siRNA sequences targeting GL2 Luciferase (Qiagen). After 72 h, protein levels in total cell lysates were analyzed by western blotting using the appropriate antibodies or the effect of the corresponding knockdown was assayed.

### Cell transfection with vectors

Cells were transfected with 5 *μ*g GFP-StarD13, dominant active RhoA, or control empty control vectors using Lipfectamine LTX with Plus reagent (Invitrogen) as described by the manufacturer. Cells were incubated with the transfection complexes for 4 h then refed with DMEM supplied with 30% FBS. The experiments were carried on 24 h following transfection. The GFP-StarD13 and the RhoA constructs were generous gifts from respectively Dr Hitoshi Yagisawa from the University of Hyogo, Japan and Dr Hideki Yamaguchi from the Albert Einstein College of Medicine, NY, USA.

The constructs were transformed into One Shot TOP10 chemically competent *E. coli* (Invitrogen), which were grown on a selective medium containing the appropriate antibiotic. The vectors were then extracted using MaxiPrep plasmid extraction kit from Qiagen. The mCherry-tagged RhoA-DA construct was a generous gift from Dr Louis Hodgson from Albert Einstein College of Medicine Yeshiva University, NY, USA.

### Western blotting

Cell lysates were prepared by scraping the cells in a sample buffer consisted of 4% SDS, 10% β-mercaptoethanol, 20% glycerol, 0.004% bromophenol blue, and 0.125 M Tris-HCl at a pH 6.8. The resulting lysates were boiled for 5 min. Protein samples were separated by SDS-PAGE on 8% (for StarD13) or 15% (for RhoA and Rac) gels and transferred to PVDF membranes overnight at 30 V. The membranes were then blocked with 5% non-fat dry milk in PBS containing 0.1% Tween-20 for 1 h at room temperature and incubated with primary antibody at a concentration of 1:100 for 2 h at room temperature. After the incubation with the primary antibody, the membranes were washed and incubated with secondary antibody at a concentration of 1:1,000 for 1 h at room temperature. The membranes were then washed, and the bands visualized by treating the membranes with western blotting chemiluminescent reagent ECL (GE Healthcare). The results were obtained on X-ray film (Agfa Healthcare). The levels of protein expression were compared by densitometry using ImageJ software.

### RT-PCR

Cells were grown in 6-well plate at density of 1×10^6^ cells/ml and were transfected by either control or StarD13 siRNA for 72 h. Total RNA was extracted performed RNeasy extraction kit (Qiagen) according to the manufacturer’s instructions. Reverse transcriptase polymerase chain reaction (RT-PCR) was used to amplify RNA of StarD13. RNA (2 *μ*g) was converted to cDNA using the OneStep RT-PCR kit (Qiagen) as described by the manufacturer. Briefly, gene-specific primers designed to detect cDNA were obtained from TIB-MolBiol with the following sequences: forward, 5′-AGC CCCTGCCTCAAAGTATT-3′; reverse, 5′-AGCCCCTGCCTC AAAGTATT-3′. β-actin was used as a control with primers obtained from Sigma-Aldrich having the following sequences: forward, 5′-ATGAAGATCCTGACCGAGCGT-3′; reverse, 5′-AACGCAGCTCAGTAACAGT-CCG-3′. Primers were used at a final concentration of 0.6 *μ*M. Primers were added to 5X Qiagen OneStep RT-PCR buffer providing a final concentration of 2.5 mM MgCl_2_ in the reaction mix. A final concentration of 400 *μ*M of each dNTP was added along with 2.0 *μ*l/reaction of enzyme mix. Final mastermix volume was adjusted to 50 *μ*l using RNase-free water. Thermal cycler conditions, for both reverse transcription and PCR, was programmed as follows: reverse transcription at 50˚C for 30 min, initial PCR activation step at 95˚C for 15 min, followed by 25 cycles of denaturation at 94˚C for 1 min, annealing at 52˚C for 1 min and extension at 72˚C for 1 min followed by a final extension step at 72˚C for 10 min. The PCR products (10 *μ*l) were run on 0.8% agarose gel stained with ethidium bromide at 100 V for 30 min. The resulting bands were visualized under UV light and photographed. β-actin was used as a loading control.

### Antigen retrieval and immunohistochemistry

Permission for tissue collection was granted by the Committee on Human Subjects in Research (CHSR) at the Lebanese American University (approval given March 26, 2010, CHSR tracking no. NSMS26032010-1). Human breast cancer tissues different grades were provided by Dr Selim Nasser from Clemenceau Medical Center (CMC), Beirut, Lebanon. Tissue blocks were paraffin-embedded and sectioned to 8-*μ*m sections using a tissue microtome. Sections were deparaffinized in two changes of xylene 5 min each then hydrated in two changes of 95% alcohol 2 min each followed by 2 changes of 50% alcohol 2 min each. Antigen retrieval was then performed in pre-heated Citra Plus (Biogenex) solution. Tissues were then fixed with 4% paraformaldehyde for 10 min, and permeabilized with 0.5% Triton-X100 for 10 min. To decrease background fluorescence, tissues were rinsed with 0.1 M glycine then incubated with 0.1 M glycine for 10 min. For blocking, tissues were incubated 4 times with 1% BSA, 1% FBS in PBS for 5 min. Samples were stained with StarD13 primary antibody for 2 h and with a fluorophore-conjugated secondary antibody for 2 h. Tissue fluorescent images were taken using a 10X objective on a fluorescent microscope. For image analysis, all digital images were imported in ImageJ software (National Institutes of Health, MA, USA). The total fluorescence intensity of a fixed area from ≥10 different frames from each tissue was determined.

### Trypan blue exclusion method

Cells were grown in 24-well plates (growth area: 2 cm^2^) at a density of 2×10^6^ cells/ml. Depending on the experiment, cells were transfected with either StarD13 siRNA or GFP-StarD13 construct. Following treatment period, the supernatant from each well was collected, cells were washed with PBS, and the PBS washes were added to the supernatant of each well. Cells were then trypsinized and collected separately from the well contents and PBS. From each collection tube 20 *μ*l was mixed with 20 *μ*l of trypan blue, 10 *μ*l of this mixture was placed in a counting chamber under the microscope, and the number of living and dead cells was recorded accordingly. For each well, two countings were done separately, PBS washes/well supernatant and trypsinized cells. Under the microscope, dead cells appear blue, since they are permeable to trypan blue, while viable cells exclude the stain and thus appear bright. The percentage of dead cells was reported.

### Cell proliferation reagent (WST-1)

Cells were seeded in 96-well plates (growth area: 0.6 cm^2^) at a concentration of 1×10^6^ cells/ml. Depending on the experiment, cells were transfected with either StarD13 siRNA or GFP-StarD13 construct with appropriate controls. Following treatment period, 10 *μ*l of cell proliferation reagent (WST-1; Roche, Germany) was added to each well. The plates were incubated at in a humidified incubator (37˚C) in 95% air and 5% CO_2_ for 2 h. WST-1 is a tetrazolium salt that on contact with metabolically active cells is cleaved to produce formazan dye by mitochondrial dehydrogenases. Quantitation of formazan is done colorimetrically at 450 nm. The absorbance of the each blank well was subtracted from the corresponding sample well. The results were normalized to the corresponding controls, and the percent of cell proliferation was reported.

### Pull-down assay

Cells were either transfected with GFP-StarD13 construct or an empty GFP construct as a control. Following treatment period, cells were lysed and the pull-down assay performed using the RhoA/Rac1/Cdc42 Activation Assay Combo Kit (Cell BioLabs) following the manufacturer’s instructions. Briefly, cell lysates were incubated with GST-RBD (for RhoA) or GST-PAK (for Rac1/Cdc42) for 1 h at 4˚C with gentle agitation. Then, the samples were centrifuged, and the pellet washed for several times. After the last wash, the pellets were resuspended with sample buffer and boiled for 5 min. GTP-RhoA and GTP-Rac1/Cdc42 were detected by western blotting using anti-RhoA, anti-Rac1 and anti-Cdc42 antibodies provided in the kit. Total proteins were collected prior to the incubation with GST beads and used as a loading control.

### Cell cycle analysis

Treated cells were placed into 15 ml Falcon tubes and centrifuged at 1,500 rpm for 5 min. The pellet was then washed and resuspended in 1 ml of ice-cold 1X phosphate-buffered saline (PBS) followed by 4 ml of 70% ethanol. Cells were then left overnight at −20˚C. The following day, cells were pelleted and washed with 1X PBS. The pellet was resuspended in 500 *μ*l of 1X binding buffer and then stained with 10 *μ*l of propidium iodide (PI) for 10 min in the dark. Cells were analyzed using an Accuri C6 flow cytometer (Ann Arbor, MI, USA), which indicated the distribution of the cells into their respective cell cycle phases based on their DNA content determined by the CFlow^®^ software. G0/G1 cells were 2n, S-phase cells were >2n but <4n while G2/M cells were 4n.

### Immunostaining assay

The cells were plated on cover slips, and the appropriate treatment was applied. Cells were fixed with 4% paraformaldehyde for 10 min, and permeabilized with 0.5% Triton-X100 for 10 min. To decrease background fluorescence, cells were rinsed with 0.1 M glycine then incubated with 0.1 M glycine for 10 min. For blocking, cells were incubated 4 times with 1% BSA, 1% FBS in PBS for 5 min. Samples were stained with primary antibodies for 2 h and with fluorophore-conjugated secondary antibodies for 2 h. Fluorescent images were taken using a 60X objective on a fluorescent microscope. Average adhesion size was obtained by thresholding the image on ImageJ software that calculates the average area and size.

### Wound healing

Cells were grown to confluence on culture plates and a wound was made in the monolayer with a sterile pipette tip. After wounding, the cells were washed twice with PBS to remove debris and new medium was added. Phase-contrast images of the wounded area were taken at 0 and 16 h after wounding. Wound widths were measured at 11 different points for each wound, and the average rate of wound closure was calculated in *μ*m/h using the ImageJ software in pixels/h and then converted to *μ*m/h by multiplying by the the pixel size corresponding the objective used in these experiments.

### Motility assay

For motility analysis, images of cells moving randomly in serum were collected every 60 sec for 2 h using a 20X objective. During imaging, the temperature was controlled using a Nikon heating stage which was set at 37˚C. The medium was buffered using HEPES and overlayed with mineral oil. The speed of cell movement was quantified using the ROI tracker plugin in the ImageJ software, which was used to calculate the total distance travelled by individual cells. The speed is then calculated by dividing this distance by the time (120 min) and reported in *μ*m/min. The speed of ≥15 cells for each condition was calculated. The net distance travelled by the cell was calculated by measuring the distance travelled between the first and the last frames.

### Adhesion assay

96-well plates were coated with collagen using Collagen Solution, Type I from rat tail (Sigma) overnight at 37˚C then washed with washing buffer (0.1% BSA in DMEM). The plates were then blocked with 0.5% BSA in DMEM at 37˚C in a CO_2_ incubator for 1 h. This was followed by washing the plates and chilling them on ice. Meanwhile, the cells were trypsinized and counted to 4×10^5^ cell/ml. Cells (50 *μ*l) were added in each well and incubated at 37˚C in a CO_2_ incubator for 30 min. The plates were then shaken and washed 3 times. Cells were then fixed with 4% paraformaldehyde at room temperature for 10 min, washed, and stained with crystal violet (5 mg/ml in 2% ethanol) for 10 min. Following the staining with crystal violet, the plates were washed extensively with water, and left to dry completely. Crystal violet was solubilized by incubating the cells with 2% SDS for 30 min. The absorption of the plates was read at 550 *μ*m using a plate reader.

### Invasion assay

MDA-MB-231 cells were transfected with either control or StarD13 siRNAs and invasion assay was performed 48 h following treatment period using the collagen-based invasion assay (Millipore) according to the manufacturer’s instructions. Briefly, 24 h prior to assay, cells were starved with serum-free medium. Cells were harvested, centrifuged and then resuspended in quenching medium (without serum). Cells were then brought to a concentration of 1×10^6^ cells/ml. In the meantime, inserts were prewarmed with 300 *μ*l of serum-free medium for 30 min at room temperature. After rehydration, 250 *μ*l of media was removed from inserts and 250 *μ*l of cell suspension was added. Inserts were then placed in a 24-well plate, and 500 *μ*l of complete media (with 10% serum) was added to the lower wells. Plates were incubated for 24 h at 37˚C in a CO_2_ incubator. Following incubation period, inserts were stained for 20 min at room temperature with 400 *μ*l of cell stain provided with the kit. Stain was then extracted with extraction buffer (also provided). Extracted stain (100 *μ*l) was then transferred to a 96-well plate suitable for colorimetric measurement using a plate reader. Optical density was then measured at 560 *μ*m.

### Statistical analysis

All the results reported represent average values from three independent experiments. The error estimates are given as ± SEM. The p-values were calculated by t-tests or chi-square tests depending on the experiment using the VassarStats: Website for Statistical Computation (http://vassarstats.net/).

## Results

### Level of expression of StarD13 in breast cancer

**Figure 1.**
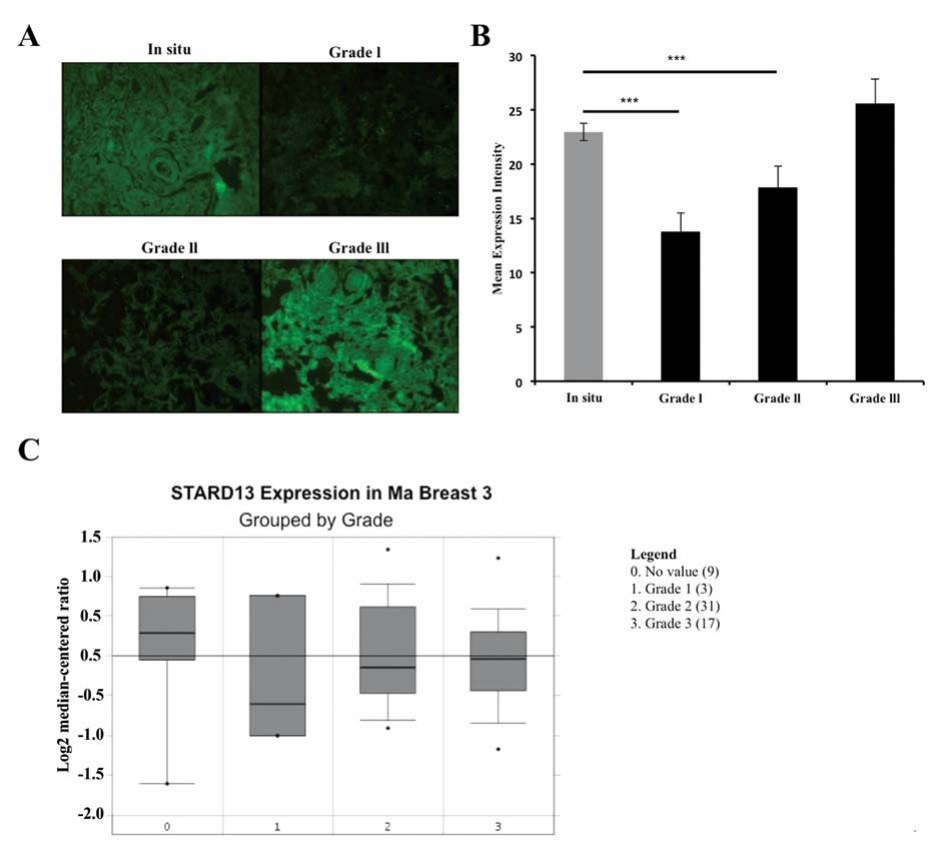
StarD13 expression levels in different grades of breast cancer. (A) Representative fluorescent micrographs of formalin-fixed breast cancer tissues were paraffin-embedded and sectioned and then immunostained with anti-StarD113 antibody: *in situ* (upper left), grade I (upper right), grade II (lower left) and grade III (lower right). Quantitation of the immunohistochemistry in (A). The mean fluorescent intensity/pixel was measured and expressed to the corresponding tissues. (B) Data are the mean ± SEM from 3 different experiments (with 5 tissues each). ^*^p<0.0001, ^**^p<0.04. (C) Data analyzed from Oncomine website. mRNA of 60 samples were quantified for expression levels.

Before studying the role of StarD13 in breast cancer cells, we first wanted to investigate its level of expression in human breast cancer tissues. For this, breast cancer tissue sections were obtained from patients representing different grades. We performed immunohistochemistry using an anti-StarD13 antibody (Fig. 1A). The mean fluorescent intensity was then measured using the ImageJ software. StarD13 showed a high expression level in non-invasive *in situ* carcinoma. Then, its level of expression decreased in grades I and II; however, as we moved on to higher grades of the tumor, StarD13 showed a significant increase in its level of expression (Fig. 1B).

In order to supplement our results, we mined the oncomine database for microarray analysis where they measured StarD13 mRNA expression levels from 60 breast cancer samples grouped by grade. The results showed that StarD13 is underexpressed in tumor tissues relative to non-tumor (grade 0). Moreover, StarD13 mRNA levels are relatively higher when compared to levels in lower grade tumors 1 and 2 (Fig. 1C). This was consistent with our IHC results.

### StarD13 effect on cell viability and proliferation

**Figure 2.**
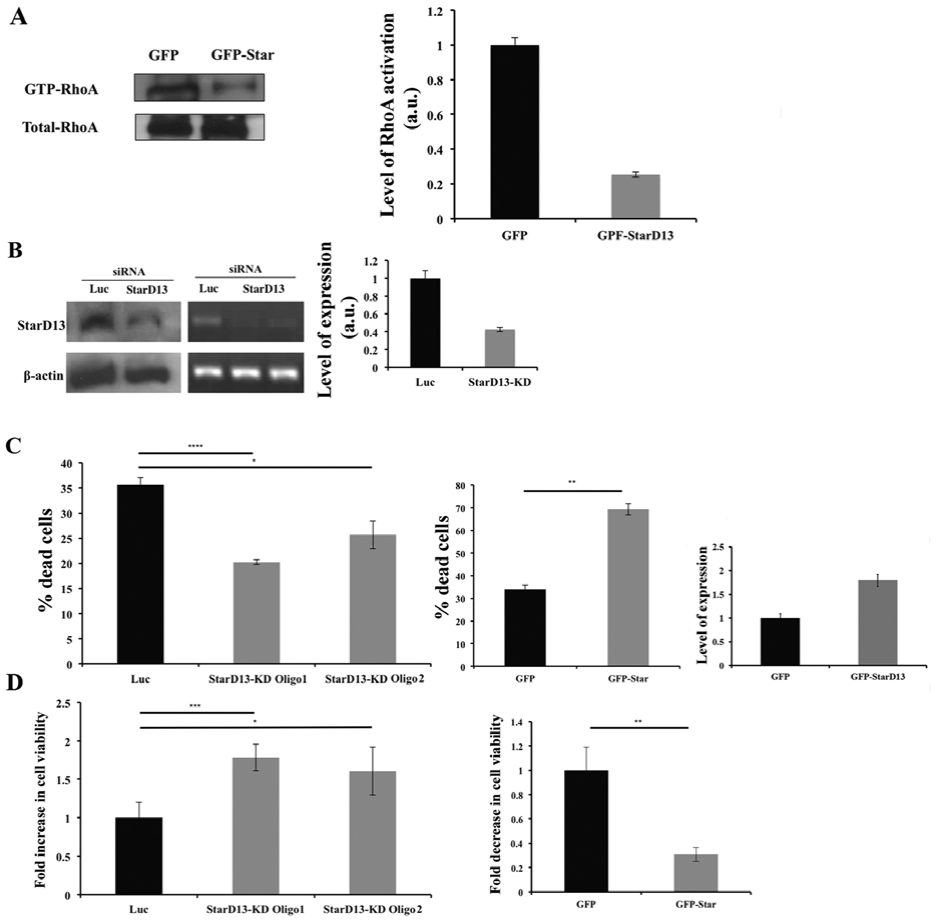
StarD13 on cell viability and proliferation. (A) MDA-MB-231 cells were transfected with either GFP alone (right lanes) or with GFP-StarD13 (left lanes). The cells were then lyzed and incubated with GST-RBD (Rhotekin binding domain) (upper pannels), or with GST-CRIB (Cdc42 and Rac interactive binding domain) (lower panels) to pull down active Rho and Cdc42, respectively. The samples were then blotted with Rho, and Cdc42 antibodies. The lower gels in each panel are western blots for the total cell lysates for loading control. The bands from the active RhoA gel were quantitated using ImageJ and normalized to the amount of total proteins. Data are the mean ± SEM of 3 blots from 3 independent experiments. (B) MDA-MB-231 cells were transfected with luciferase control siRNA or with StarD13 siRNA for 72 h. Two different siRNA oligos against StarD13 were used in each experiment. The cells were lysed and immunoblotted by western blot analysis for StarD13 (left upper gel) or for actin (left lower gel) for loading control. Quantitation represents 3 different blots from independent experiments. RT-PCR was also performed to detect StarD13 level using StarD13 primer (right upper gel) and actin as loading control (right lower gel). (C) Percentage (%) of dead cells was determined using trypan blue, results are shown as percent of total number of cells (left panel). Cells were transfected with GFP-StarD13 or GFP alone as control for 24 h. The percentage of dead cells showed an increase of 50% as determined using trypan blue (middle panel). Right panel is a quantitation from 3 different blots of cells transfected with either GFP or GFP-Stard13 and blotted for Star. (D) Cell proliferation was determined using WST-1 reagent. Cell viability of siRNA-transfected cells was expressed as fold increase from control (right panel). Cell proliferation dramatically decreased in cells transfected cells with GFP-StarD13 construct as opposed to control cells (right panel). Data are the mean ± SEM from 3 different experiments. ^*^p<0.02, ^***^p<0.001, ^****^p<0.0001.

The surprising increase in StarD13 expression level in higher grades of breast cancer lead us to investigate whether StarD13 is indeed a tumor suppressor in our cells. Next, we wanted to investigate the role of StarD13 on cellular proliferation and viability. After confirming its GAP activity on RhoA and Cdc42 (Fig. 2A) by pull-down assay, we looked at the effect of StarD13 on proliferation. StarD13 was knocked down using small interfering siRNA in MDA-MB-231 cells. The resulting inhibition in the level of StarD13 was determined using western blotting and RT-PCR in cells transfected with 2 different StarD13 siRNA oligos as compared to control cells transfected with non-specific siRNA where β-actin was used as a loading control (Fig. 2B). A 60% knockdown has been observed in western blotting (Fig. 2B). StarD13 knockdown resulted in a 40% decrease of dead cells as determined by trypan blue exclusion method (Fig. 2C).

On the other hand, as a second approach, cells were transfected with a GFP-StarD13 construct and the resulting cell viability was determined as compared to cells transfected with GFP vector alone. Cells overexpressing StarD13 showed a drastic increase in the percentage of dead cells as compared to control cells as determined using trypan blue (Fig. 2C).

Moreover, this was reflected as an increase in cell viability in cells transfected with StarD13 siRNA by 18% as opposed to control cells as determined using Wst-1 reagent. In contrary, there was a dramatic decrease in cell viability in cells overexpressing StarD13 (Fig. 2D).

### StarD13 effect on the cell cycle

**Figure 3.**
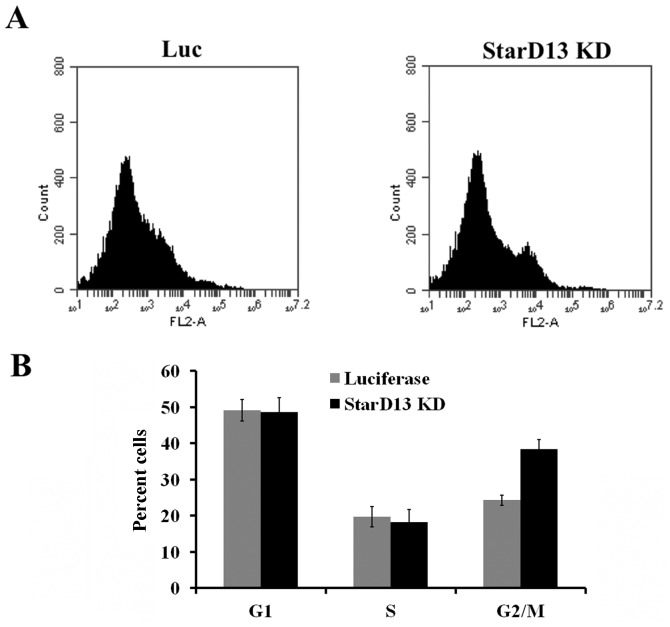
StarD13 effect on the cell cycle. (A) MDA-MB-231 cells transfected with either control luciferase or StarD13 siRNA for 72 h. Cells were fixed overnight and stained with 10 *μ*l of propidium iodide (PI). Cells were analyzed using a C6 flow cytometer, which indicated the distribution of the cells into their respective cell cycle phases based on their DNA content. G0/G1 cells were 2n, S-phase cells were >2n but <4n while G2/M cells were 4n. (B) Quantitation of (A). Data are the mean ± SEM from 3 different experiments.

Analysis of the cell cycle where cells were stained with propidium iodide alone showed that upon StarD13 knockdown, cells seem to undergo more cell division. This is apparent in the increase in the percentage of cells in the G2/M phase (38%) as opposed to control cells (28%) (Fig. 3).

### StarD13 is needed for breast cancer cell motility

**Figure 4.**
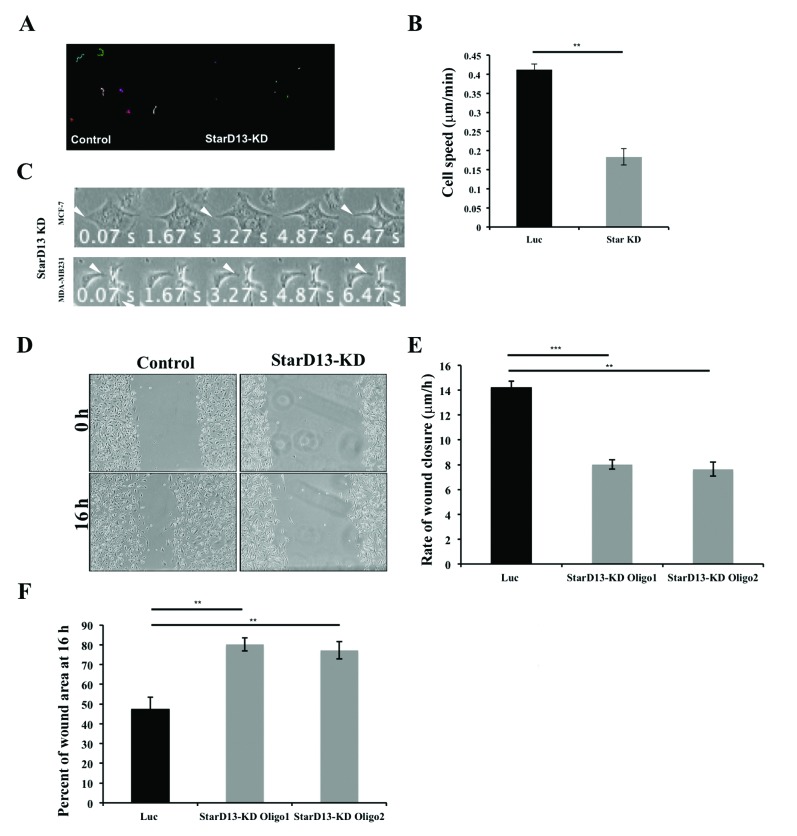
StarD13 is needed for breast cancer cell motility. Cells were transfected with luciferase control siRNA or with StarD13 siRNA for 72 h. (A) The net paths of projected 120 frames from 2 h long time lapse movies of cells undergoing random motility in serum (upper right panel) (B) Quantitation of the cell speed from expressed in *μ*m/min. Data are the mean ± SEM from n=20 cells. The results were significant with ^**^p<0.001. (C) Montage of time-lapse movie (60 sec apart) showing StarD13 siRNA-transfected cells undergoing random motility in serum (MCF-7, upper panel; and MDAs, lower panel). (D) MDA-MB-231 cells were grown in a monolayer then wounded and left to recover the wound then imaged at the same frame after 16 h (lower micrographs). (E) Quantitation of wound widths were measured at 11 different points for each wound, and the average rate of wound closure for the luciferase and the StarD13 siRNA-transfected cells was calculated in *μ*m/h (lower right panel). Data are the mean ± SEM from 3 wound closure assays from 3 independent experiments. The results were significant with ^**^p<0.02, ^***^p<0.001. (F) The percentage of wound area at 16 h following wounding. Data are the mean ± SEM from 3 wound closure assays from 3 independent experiments. The results were significant with ^**^p<0.002.

We were then interested in investigating the role of StarD13 in breast cancer cell motility. For this reason, StarD13 was knocked down using siRNA oligonucleotides (Fig. 2A). Results show that StarD13 knockdown significantly decreased the average speed of individual cells from 0.41 to 0.20 *μ*m/min as determined by time-lapse motility movies (Fig. 4B). Looking at the morphology, both cell lines MCF-7 and MDA-MB-231 were observed to be stuck and not able to detach their tail in order to move forward (Fig. 4C). Moreover, StarD13 knockdown decreased the rate of wound closure from 14 *μ*m/h to ∼7 *μ*m/h in both oligos tested (Fig. 4D and E). Also, the area of the wounds were calculated at 16 h following the formation of the wounds. The results show that in control cells only 47% of the initial wound area is left as opposed to StarD13 knockdown cells where the wounds areas did not appear to change after 16 h, 80% for oligo1 and 77.16% for oligo2 (Fig. 4F). These results indicate that the knockdown of StarD13 inhibits breast cancer cell motility.

### Regulation of RhoA and Rac activations is necessary for cell movement

**Figure 5.**
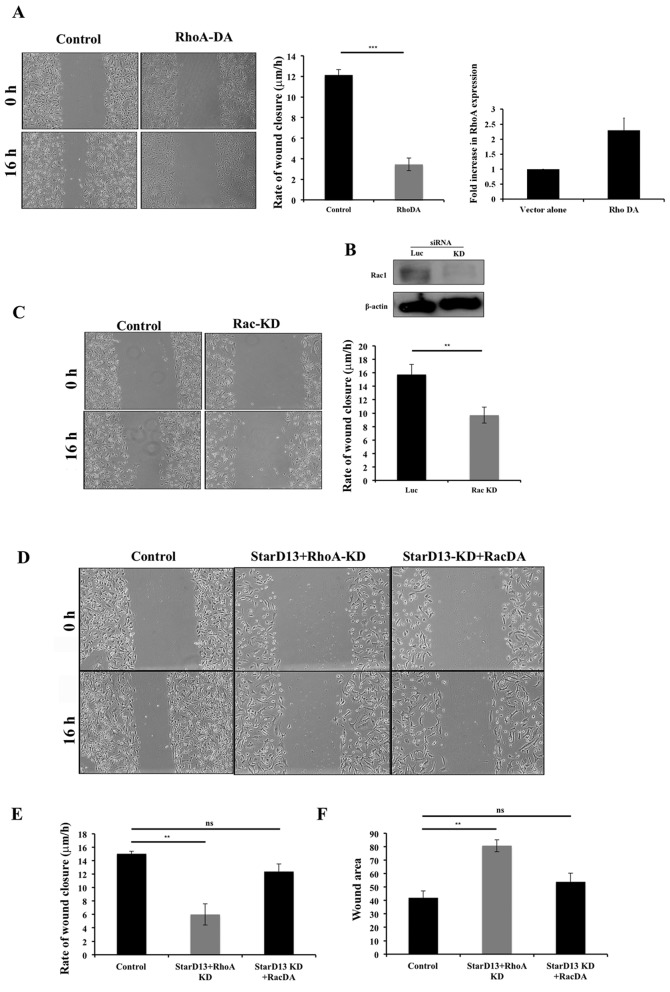
Constitutive activation of RhoA inhibits cell motility. (A) MDA-MB-231 cells were left untransfected or transfected with a dominant active RhoA construct (RhoA DA). Cells were grown in a monolayer then wounded and left to recover the wound then imaged at the same frame after 16 h (lower micrographs). Wound widths were measured at 11 different points for each wound, and the average rate of wound closure was calculated in *μ*m/hr. Data are the mean ± SEM from 3 wound healing assays from 3 independent experiments. The results were significant with ^***^p<0.001. Right panel, cells were transfected as described, lysed and immunoblotted for RhoA. The gels were quantitated and data are the mean ± SEM from 3 different gels. (B) Western blot showing the decrease in Rac1 expression compared to actin (lower panel) in cells transfected with Rac1 siRNA compared to luciferase control (left lane). MDA-MB-231 cells were grown in a monolayer then wounded and left to recover the wound then imaged at the same frame after 16 h (lower micrographs). Quantitation of wound widths were measured at 11 different points for each wound. (C) The average rate of wound closure for the luciferase and the Rac1 siRNA-transfected cells was calculated in *μ*m/hr. Data are the mean ± SEM from 3 wound closure assays from 3 independent experiments. The results were significant with ^**^p<0.01. (D) MDA-MB-231 cells were transfected with both StarD13-siRNA and RhoA-siRNA double knockdown or both StarD13-siRNA and Rac1-DA construct. Cells were grown in a monolayer then wounded and left to recover the wound then imaged at the same frame after 16 h. (E) The average rate of wound closure for the control and transfected cells was calculated in *μ*m/hr. Data are the mean ± SEM from 3 wound closure assays from 3 independent experiments. The results were significant for the StarD13+RhoA-KD with ^**^p<0.002 and not significant (ns) for StarD13KD+RacDA. (F) The percentage of wound area at 16 h following wounding. Data are the mean ± SEM from 3 wound closure assays from 3 independent experiments. The results were significant for the StarD13+RhoA-KD with ^**^p<0.002 and not significant (ns) for StarD13KD+RacDA.

After showing that RhoA knockdown inhibits cell motility (data not shown) and that StarD13 knockdown, where RhoA is kept active, also inhibits cell motility (Fig. 4) it was of great interest to us to determine the effect of overexpressing a constitutively active form of RhoA. Thus, similar to StarD13 knockdown, dominant active RhoA suppressed cellular motility. This was observed through wound healing assay where the rate of wound closure was decreased from 12.3 to 3.4 *μ*m/h (Fig. 5A). Knowing that Rac1 plays a major role in breast cancer motility and adhesion formation and knowing the antagonistic effect of Rho and Rac (20), we also opted to investigate the role of Rac1 in breast cancer cell motility. For this purpose, Rac1 was knocked down using a specific siRNA. The resulting expression level was detected by western blotting in cells transfected with Rac1 siRNA as compared to control cells transfected with non-specific siRNA (Fig. 5B). Results showed a decrease in the rate of wound closure in cells transfected with Rac1 siRNA to 9.4 *μ*m/h as compared to control cells (15.7 *μ*m/h) (Fig. 5C). Since StarD13 knockdown results in continuous activation of RhoA, we next looked at the effect StarD13 and RhoA double knockdown. In this case, the rate of wound closure observed was 6 *μ*m/h (Fig. 5D and E) and almost 80% of the wound area remained after 16 h of wound formation (Fig. 5F). We also looked at the effect of StarD13 knockdown coupled with the expression of constitutively activated Rac1. This resulted in a 12.39 *μ*m/h rate of wound closure as compared to 15.05 *μ*m/h in control cells, thus indicating a rescuing effect.

### StarD13 exerts its effects on RhoA and Rac in focal adhesions

**Figure 6.**
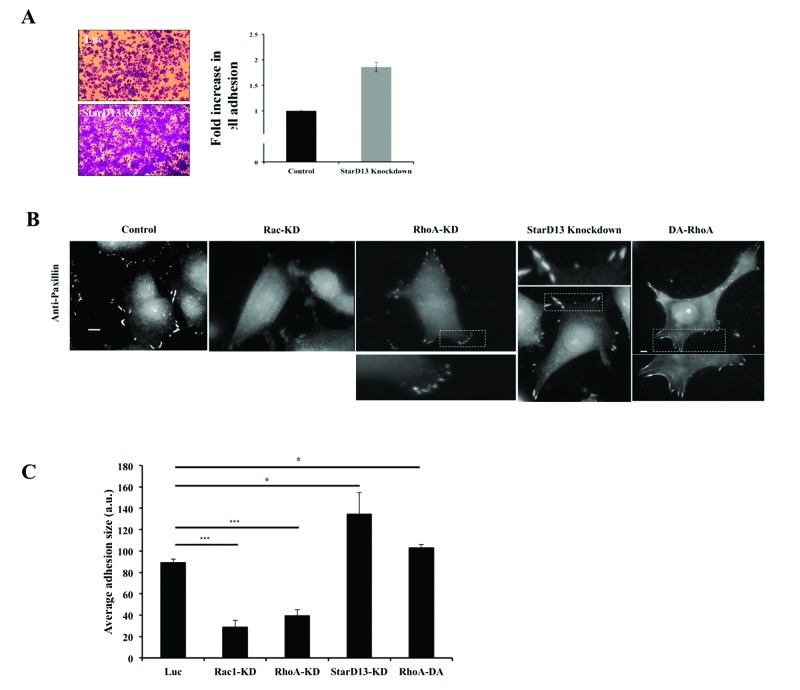
StarD13 exerts its effects on RhoA and Rac in focal adhesions. (A) Representative micrographs of cells fixed and stained with crystal violet to detect adhesion (as described in Materials and methods). Crystal violet was solubilized and the absorption of the plates was read at 550 *μ*m using an ELISA plate reader. Data were measured in arbitrary units and normalized to the luciferase control. Data are the mean ± SEM from 3 experiments. The results were significant with p<0.001. (B) Representative micrographs of (MCF-7) cells that were transfected with either luciferase, Rac siRNA RhoA siRNA, StarD13 siRNA and fixed and immunostained with anti-paxillin antibody. Cells that were transfected with RhoA-DA construct fixed and immunostained with anti-paxillin antibody. Cells were imaged using a 60X objective. (C) Quantitation represented as average adhesion size in control cells and in cells with Rac, RhoA and StarD13 knockdown and in cells expressing RhoDA. Data are the mean ± SEM from n=15 cells. The results were significant with ^***^p<0.0001, ^*^p<0.05. Scale bar represents 10 *μ*m.

Looking at the effect of StarD13 knockdown on the adhesion of breast cancer cells to collagen, our results show that cells with StarD13 knockdown have increased adhesion to collagen by >2-fold as compared to control cells (Fig. 6A).

After showing the effect of StarD13 on cell motility, we were interested in looking directly at focal adhesion under conditions where cells are deficient in Rac1 levels as well RhoA and StarD13. For this reason, we immunostained for Paxillin, a component of both focal complexes and focal adhesions (17) using anti-paxillin antibody.

In cells with Rac1 knockdown, neither focal complexes nor focal adhesions were visible at the cell edge (Fig. 6B), which is correlated with reduced migration (Fig. 5C). This is in accordance with previous studies that have shown that Rac1 is needed for the initial formation of focal contacts (22). In cells with RhoA knockdown, focal adhesions were less prevalent compared to control cells; instead small punctate structures were highly present representing immature focal complexes (Fig. 6C). This was reflected in a 67% reduction in the average adhesion size as compared to control cells in the case of Rac1-knockdown and 55% reduction in cells with RhoA knockdown (Fig. 6C). In cells with StarD13-KD; however, focal adhesions were more prominent and more pronounced especially at the cell edges as compared to control cells (Fig. 6B). Quantitatively, the average adhesion increased by ∼34% compared to control cells (Fig. 6C). Consistent with our StarD13 siRNA knockdown, we found that transfecting the cells with a dominant active form of RhoA lead to the stabilization of focal adhesions (Fig. 6B). This was also reflected by a 13.5% increase in average adhesion size in cells transfected with a dominant active form of RhoA as compared to control cells (Fig. 6C).

### StarD13 increases cellular invasion

**Figure 7.**
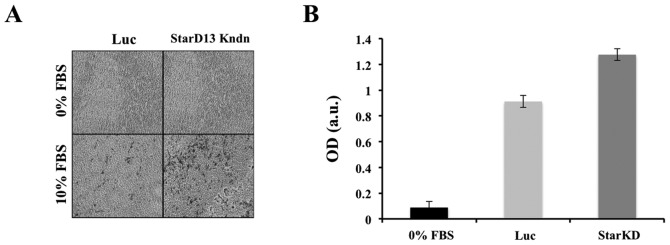
StarD13 knockdown increases cellular invasion. (A) Representative micrographs of invaded cells on the bottom side of the collagen-coated membrane stained with cell stain according to assay instructions. MDA-MB-231 cells with StarD13 knockdown and control cells were allowed to invade towards 10% FBS for 24 h. Cells/ml (1×10^6^) were used in each assay. (B) Cell stain was extracted and colorimetric measurements were taken at 560 *μ*m. Data are measured in arbitrary units.

After establishing the role of StarD13 in 2D cell migration, we were interested in determining its role in 3D cell invasion. For this reason, we performed an *in vitro* collagen-based invasion assay using FBS as a chemo-attractant. A chamber with serum-free media in both wells was used as negative control. To our surprise, unlike its effect in 2D, there was nearly a half-fold increase in cell invasion in cells with StarD13 knockdown as compared to control cells (Fig. 7).

### Proposed model for StarD13 regulation

Our results showed that StarD13 negatively affects cellular proliferation. However, having a RhoGAP activity and localizing to focal adhesions of the cell, we showed that StarD13 actually plays an essential role in cellular motility. This correlated to the increase in its expression in metastatic forms of the tumor. In this context, looking at the dynamics of focal adhesion, StarD13 seemed to be involved in the inhibition of RhoA following the maturation of FAs that results in the detachment and forward movement of the cell. Upon silencing StarD13 using siRNA, cells showed elongated tail morphology with stabilized focal adhesions and inhibitory cellular motility. However, this was not correlated in 3D mode, where we saw that cells with StarD13 knockdown seemed to have an enhanced invasive ability.

## Discussion

In the present study, we opted to characterize StarD13 in breast cancer in terms of expression, effect on cell proliferation and viability, GAP activity and role in motility and invasion. Previous studies by Ching *et al* (12) identified StarD13 as a tumor suppressor gene in hepatocellular carcinoma cells. In the present study, we examined the role of StarD13 in breast cancer cell proliferation and motility. Looking at its level of expression by IHC we revealed that StarD13 is highly expressed in non-tumor *in situ* form of breast cancer and it is downregulated in grades I and II, suggesting the potential role of StarD13 as a tumor suppressor in breast cancer. This was in accordance with previous studies where StarD13 was found to be underexpressed in several cancer types including lung, colon, gastric, ovarian, uterine, renal and rectal tumors (13). However, StarD13 showed a relatively high expression in highly metastatic forms of breast cancer. This was supplemented by data obtained from Oncomine database in which mRNA levels of StarD13 were reduced in grades I and II as compared to normal, however, were shown to increase in higher grades of the tumor. This was in accordance with a previous study in astrocytoma where StarD13 was shown to be overexpressed in grades III and IV as compared to grades I and II of the tumor (16).

We also showed that StarD13 has an anti-proliferative effect on breast cancer cells. This was evident when the overexpression of StarD13 dramatically increased cell death. In contrast, silencing StarD13 using specific siRNAs led to a decrease in cell death and an increase in cellular viability. Although no effect was apparent on apoptosis upon knocking down StarD13 these cells, further analysis of the cell cycle showed that silencing StarD13 lead to slight increase in dividing cells. This is consistent with its role as a tumor suppressor. Therefore, consistent with the literature, StarD13 seems to function as a tumor suppressor in breast cancer.

While examining the role of StarD13 in cell motility, we found that the knockdown of StarD13 in breast cancer cell lines inhibited cell motility. This may explain the increase of StarD13 expression in grades III obtained in IHC. Hence although being a tumor suppressor, this protein is needed for motility. Indeed, looking at their morphology, StarD13 knockdown cells were immobilized and unable to detach their tail in order to retract their cell body and move forward. This was also reflected by an increase in cellular attachment through the stabilization of focal adhesions.

RhoA has been extensively proven to be indispensable for the formation of focal adhesions and for cell motility in several systems (18,19). Our data show that knockdown of RhoA leads to inhibition of cellular motility. As a RhoA-GAP, StarD13 knockdown is hypothesized to result in continuous activation of RhoA. This surprisingly also led to an inhibition of cell motility. StarD13 and RhoA double knockdown also resulted in a dramatic inhibition of the cells ability to migrate. These experiments show that, while required for motility, consistent RhoA activation inhibits it suggesting that RhoA needs to go through cycles of activation and inactivation. On the other hand, cells with StarD13 knockdown coupled with the expression of constitutively activated Rac1 showed increased migration ability in wound healing similar to StarD13 knockdown cells indicating a rescuing effect. This could be explained by the antagonistic relationship between RhoA and Rac. When StarD13 is knocked down, RhoA is constitutively active which inhibits Rac. This inhibits motility as Rac is also required for motility. Furthermore, it has been previously shown that increasing Rho activation inhibits the dissolution of focal adhesions at the tail of moving cells, inhibiting cell motility (20,21). Thus, we formulated the hypothesis that StarD13 knockdown is keeping RhoA active in focal adhesions at the tail leading to the inhibition of cell motility. This persistent activation of RhoA is also keeping Rac inhibited not allowing Rac to initiate focal complexes which are precursors for focal adhesions as described below.

Knowing that Rac1 plays a major role in breast cancer motility and adhesion formation and knowing the antagonistic effect of RhoA and Rac (20), we started by looking at the dynamics of cellular adhesion directly following Rac and RhoA knockdowns. In cells with Rac knockdown, neither focal complexes nor focal adhesions were observed. This is in accordance with the fact that Rac is needed for the formation of focal complexes (22). Moreover, cells underexpressing RhoA showed inability to form mature focal adhesions. Similarly previous studies done on MTLn3 cells showed that inhibition of RhoA downstream effector ROCK blocked the maturation of focal adhesions in MTLn3 cells (23). However, silencing StarD13 led to the stabilization of focal adhesions and decrease in focal complexes. Hence, we suspected that cells with StarD13 knockdown, seem to have a constitutively active RhoA stabilizing cellular adhesion to the underlying substratum and impeding tail retraction resulting in inhibition of cell motility. The disassembly of focal adhesions is required for the completion of the cell motility cycle. This suggests that StarD13 plays a role in inhibiting RhoA leading to the detachment of the cell. In this sense, overexpressing a dominant active form of RhoA would mimic StarD13 knockdown phenotype in cell motility and adhesion. Indeed, transfecting the cells with a constitutively active RhoA inhibited cell motility and cells showed larger and more abundant focal adhesions relative to focal complexes. Similar studies involving the use of dominant active RhoA have demonstrated an inhibition of cellular motility (24–26).

Taken together, our data show that even though StarD13 is known to be a tumor suppressor, it is needed for motility. This controverts traditional concepts regarding definite roles of tumor suppressors versus oncogenes in different cancer types. Studies done on *DLC1*, a closely related gene, reported its function as a candidate tumor suppressor in hepatocellular carcinoma (27). Further studies showed that hypermethylation of *DLC1* gene promoter is responsible for the loss of its function as a tumor suppressor in a subset of liver, colon and prostate cancers (15,28–30). Other studies also confirm the role of DLC1 as a tumor suppressor (29). In this framework, overexpression of DLC1 was shown to exhibit inhibitory effects on cell growth and proliferation in hepatocellular and breast cancer (28,30,31). Moreover, DLC1 activity was shown to positively regulate the cytoskeleton and ultimately cell motility. This regulatory mechanism was linked to the its negative regulation of RhoA through its GAP activity (32,33). Another recent study done on normal prostate cells showed that silencing of DLC1 reduces migration (34). In fact, recent studies showed that DLC1 plays differential roles in regulating cell migration and transformation depending on its interaction with tensins (35). This highlights the differential role of the DLC family of proteins as tumor suppressors yet needed for cell motility. A comparable dilemma is illustrated in a recent review on TGF-β that is known to exert tumor-suppressive effects in normal cells yet paradoxically, in protumorigenic cells its role is reversed (36).

After determining the mechanism by which StarD13 might affect random 2D cell motility, it was intriguing to study its effect on cellular invasion in 3D. For this we transfected the cells with siRNA against starD13 and performed collagen-based transwell invasion assay. Knowing that StarD13 knockdown inhibited cellular motility in 2D it was assumed that it would also inhibit cell invasion. However, to our ultimate surprise, silencing StarD13 had a positive effect on cellular invasion, despite the fact that StarD13 knockdown stabilizes focal adhesions. This might be due to focal adhesions playing an unconventional role in cellular invasion. A recent report exists on the contribution of focal adhesions to matrix degradation. Results revealed that several cell lines degraded underlying ECM specifically at focal adhesion sites. This process occurred through proteolytic activity of MMPs and not due to physical tension exerted by FAs onto the matrix (37). Moreover, other studies demonstrated that silencing RhoA leads to the inhibition of cellular invasion, particularly in breast cancer cell lines (38,39). This solidifies our data with starD13 knockdown where we typically have an increase in RhoA activity, thus promoting cellular invasion. Moreover, it was previously discovered that in 3D matrices, tumor cells are able to switch between distinct modes of motility (40). This pertains to the interplay between different signaling requirements. Thus, cells can switch between a rounded blebbing movement and a more elongated protrusive fashion. Thus in our study, the depletion of StarD13 increased cellular adhesion to the ECM impeding 2D mesenchymal cellular migration; however, this was reflected in an increase in 3D movement. This suggests that when cells cannot move in an adhesion-dependent manner, they tend to switch to a more amoeboid fashion. Therefore, the ability of tumor cells to switch between modes of motility may limit the effectiveness of prospective inhibitory strategies targeting particular cell morphology, hence promoting the selection of a different mode to escape inhibition.

We conclude that StarD13 tumor suppressor activity is contextual where tumor cells manage to exploit alternative mechanisms to escape inhibition. This reveals the importance of understanding the complexity and diversity of these pathways as a tool in paving the way for finding potential therapeutic targets.
